# A Multi-Criteria Framework for the Sustainable Management of Fisheries: a Case Study of UK’s North Sea Scottish Fisheries

**DOI:** 10.1007/s00267-022-01607-w

**Published:** 2022-03-15

**Authors:** Negar Akbari, Trond Bjørndal, Pierre Failler, Andy Forse, Marc H. Taylor, Benjamin Drakeford

**Affiliations:** 1 Operations and Systems Management, Faculty of Business and Law, University of Portsmouth, Richmond Building, Portland Street, Portsmouth, PO1 3DE UK; 2 SNF Centre for Applied Research at NHH, Hellevn 30, N-5045 Bergen, Norway and Nord University Business School, N-8206 Bodø, Norway; 3 Centre for Blue Governance, Faculty of Business and Law, University of Portsmouth, Richmond Building, Portland Street, Portsmouth, PO1 3DE UK; 4 Thuenen Institute of Sea Fisheries, Herwigstraße 31, 27572 Bremerhaven, Germany

**Keywords:** Sustainable Fisheries Management, Multi-criteria analysis, Scottish fisheries, Brexit, North Sea

## Abstract

In this paper, a sustainability framework with a case application for UK’s Scottish fisheries has been developed which integrates aspects related to economic growth, social development, governance, biology, environment, and logistics. Scotland is the centre of UK’s commercial fishery sector however it faces challenges such as overexploitation, and changes in the governance structure following Brexit. The contributions of this study are threefold including (i) collecting and analysing primary data gathered from a diverse group of stakeholders in the Scottish fishery sector and scientific community, (ii) prioritising a diverse range of criteria in terms of importance in decision making from industry and scientific community perspectives, (iii) elaboration of the key management objectives in this region within the context of sustainable management of fisheries in the UK.

The results of this stakeholders’ survey show that the key management objectives are reductions in overexploitation of stocks, inclusive governance, increase in transparency and simplicity of policy measures, reduction in marine litter, and increase in the efficiency of vessels. The analysis also shows that the industry group places a higher importance on socio-economic objectives such as increase in profit and employment compared to the scientific group. On the other hand, the scientific group prioritised the objectives such as reducing discards, bycatch, and impact on seafloor compared to the industry group. This study provides insight for the UK’s fisheries sector, and scientific advisory groups for the enhanced implementation of sustainable fisheries management policies.

## Introduction

The North Sea is one of the most productive fishing areas in the world with estimated total annual landings of 2 million tonnes, with the largest fleets coming from the United Kingdom (UK), Norway, Denmark, Netherlands, and France (ICES 2019). The UK has significant fishing interest in the North Sea with 43% of all UK vessels’ landings coming from the northern North Sea (MMO 2019) (Bjørndal and Munro 2020). Scotland is the major centre for the UK’s commercial fishing industry with large fishing hubs which are home to some of the important operators in the industry (Forse et al. 2021). A study in 2016 suggests EU vessels were landing up to 51% of all catch by weight in Scottish waters and Scotland has the fourth-largest sea area within Europe and a long history of commercial fishing, with much of the county’s past economic performance achieved through fishing (Weir and Kerr 2020).

Following the decision to leave the EU and the common fishery policy (CFP), the management of UK fisheries as well as EU fisheries will be affected, requiring new and sustainable approaches to be adopted. Future relations between the UK and the EU will be governed by the 2020 EU-UK Trade and Cooperation Agreement which includes fisheries in addition to other areas such as trade and transportation (Bjørndal and Munro 2020). To secure the long-term sustainability of the fishery sector, policy makers may elucidate trade-offs between many different aspects such as maintaining profitability and reducing environmental impact while controlling over-exploitation of stocks. Therefore, decision makers are faced with a number of different and often conflicting objectives, and prioritisation of these objectives is crucial in making effective and informed decisions for the sustainable management of fisheries.

The objective of this paper is to develop a sustainability framework within the context of sustainable management of Scottish fisheries and prioritise the sustainability objectives in terms of their importance for decision making. The multi-criteria analysis undertaken in this paper considers environmental, economic, social, governance and logistics criteria, and the importance of this set of key criteria in decision making is provided by involving a diverse range of stakeholders and conducting a survey for collecting expert judgments from the Scottish fishing sector.

In the remainder of this paper, the material and methods are presented in Section 2, followed by the results in Section 3. The discussions are presented in Section 4 and Section 5 provides the conclusions, and future research avenues.

## Materials and methods

In this section, the background of the study including a review on the state of the art, definition of criteria selected in this framework, the survey design, structure of the survey questions, and the composition of the stakeholders are provided in detail.

### Literature review

Decision-making in natural resource management requires consideration of trade-offs among various criteria and is often complicated by various stakeholder views. Sustainable management of fisheries encompassing several objectives and criteria and involving numerous stakeholders is a growing concern, and multi-criteria approaches combined with expert participation methods have been applied frequently in the literature to address fisheries management challenges (Nielsen et al. 2019) (Huang et al. 2011). In Table 1, a summary of the studies in which participatory methods for involving stakeholders have been applied is presented along with the criteria that have been used in the studies.

**Table 1:** Summary of the literature

Author	Method	Criteria	Region
(Spoors et al. 2021)	Survey with fishers	Economic, Environmental	Creel fisheries in Scotland
(Pope et al. 2019)	Participatory Multi-criteria decision approach	Environmental, Economic, Institutional	North Sea fisheries
(Nielsen et al. 2019)	Participatory decision support tool	Biological, Economic	West coast Scotland-Cod and whiting
(Romeo and Marciano 2019)	Participatory Multi-criteria approach	Biological, Economic Social	Mediterranean
(Williams et al. 2018)	Multi-attribute utility theory	Economic, Environmental, Biological, Logistics	English Chanel fisheries-European Seabass
(Rindorf et al. 2017)	Participatory Multi-criteria approach	Economic, Ecosystem, Governance, Social	European fisheries
(Morton et al. 2016)	semi-structured interviews with representative stakeholders	Social, Political	Scottish Wild Salmon fisheries
(Kempf et al. 2016)	Participatory Multi-objective approach	Governance	North Sea
(Rossetto et al. 2015)	Participatory Multi-criteria decision approach	Biological, Economic, Social	Mediterranean
(Heen et al. 2014)	Participatory Multi-criteria decision approach	Socio-Economic, Environmental	Norway-Cod fisheries
(Ross 2013)	In depth case study and interview with Fishermen	Social	North East Scotland
(Innes and Pascoe 2010)	Participatory Multi-criteria decision approach	Bycatch, Habitat damage	European fisheries
(Utne, 2008)	Participatory Multi-criteria decision approach	Socio-Economic Environmental	Norway-Cod fisheries
(Nielsen and Mathiesen 2006)	Participatory Multi-criteria decision approach	Economic, Biological, Political	Norway and Denmark fisheries
(Mardle et al. 2004)	Participatory Multi-criteria decision approach	Economic, Allocation, and Conservation	English Channel fisheries

The review of aforementioned studies shows that stakeholders’ participation and engagement are considered and stakeholders assist with weighting the importance of criteria for guiding decision making for improved fisheries management. The methods for collecting data vary in different studies and include approaches such as focus groups, Delphi method, interviews (face to face, telephone interviews), online and/or paper form surveys. The number of criteria, as well as the number of participants in the studies, vary greatly and the most often used decision criteria groups are socio-economic and biological and the number of respondents ranges between 8 (Utne 2008) up to more than 400 respondents (Kimani et al. 2020).

Following the review of the current literature, only a few studies were identified which have focused on the evaluation of the sustainable management of Scottish fisheries considering a multitude of criteria. Furthermore, the majority of studies have considered mainly biological and socio-economic aspects with other important criteria such as logistics and governance having been addressed less frequently in the literature. The governance aspect of Scottish fisheries becomes an important issue following the UK’s exit from the EU and in this study, this aspect, as well as socio-economic, environmental, logistics, and biological, have been taken into account.

The proposed framework in this paper is developed by collecting primary data through a survey of a diverse range of stakeholders including academia, scientific advisory organisations, industry representatives, producers, fishermen, and vessel owners. The aim of involving stakeholders and managers is to ensure that the policies respond to the operational management problems of the users (Macher et al. 2018). This paper aims to strengthen the science-industry interface for improved decision-making in the Scottish North Sea fisheries through incorporating different stakeholders’ viewpoints. This approach enables policy makers to reach a consensus on the needs and priorities for the sustainable management of fisheries and to link these priorities into a broader fisheries policy framework.

### Definition of criteria

The global scientific awareness of long-term threats to vulnerable ecosystems has called for the development of sustainability science (Sala et al. 2015). As a consequence, sustainable development has gained universal appeal since it strives to achieve a harmonisation between economic growth and environmental concerns (Munda, 2016). In classical fisheries science, sustainability often refers to the catch levels that could be maintained over time (Maximum Sustainable Yield) or static or dynamic maximum economic yield (MEY) (Bjørndal and Munro 2012), i.e., the conservation paradigm of sustainable fisheries with focus on maximising the productivity of a given stock in terms of catch or economic returns. However, fisheries management in many jurisdictions including Scotland may also pursue additional objectives. These objectives may include social objectives such as maintaining employment, economic objectives such as ensuring profitability, governance objectives such as ensuring flexibility or minimising management transaction costs, as well as conservation objectives such as reducing the impact of fishing on key habitats (Rindorf et al. 2017). Hence a one-dimensional MSY or MEY approach may not be sustainable over time since it may not take into account an integrated approach to fisheries management. Furthermore, the role of fisheries is often ignored such that the fishermen’s low influence in the decision making process leads to their marginalisation and may lead to ineffective policy interventions (Semitiel-García and Noguera-Méndez, 2019).

**Fig. 1:**
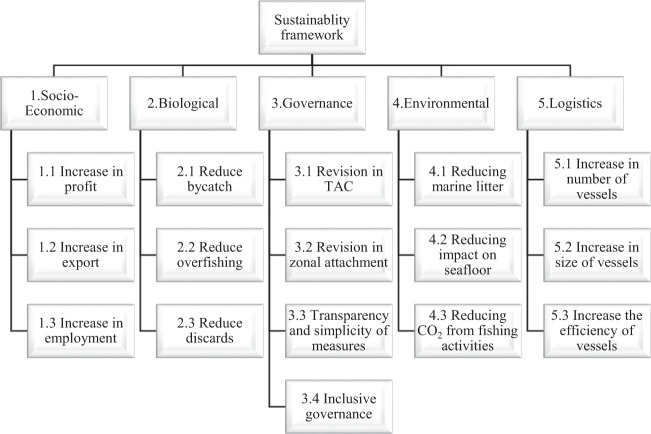
Criteria hierarchy for sustainable management of fisheries

In a framework designed by (Anderson et al. 2015), it is proposed that fisheries management should achieve three objectives of economic, environment and community sustainability. Based on this framework, we define five main groups of sustainability criteria including socio-economic, governance, biological, logistics and environmental category along with a number of sub-criteria as shown in Fig. 1. These criteria (indicators) support decision making and enable setting objectives in fisheries including internal management (stakeholders) and external assessment (government organizations). In the remainder of this section, a description of these criteria within the context of Scottish fisheries is provided.

#### Socio-economic

Socio-economic aspect is concerned with the economic performance of fisheries and regional employment created by the sector. In Scotland, public investment in fishery-related infrastructure appears to have been consolidated around strategically important ports such Peterhead and Fraserburgh, leading to smaller ports missing out on investment opportunities and hence lower socio-economic development. In terms of workforce-by 2019- in the catching sector, approximately 27% of the overall workforce were non-UK nationals, of which approximately 8% were from the European Economic Area (EEA) and 19% are from non-EEA nationals (the majority of which are from the Philippines).

For the seafood processing sector, an important consideration following EU exit is maintaining free movement of labour from the EU. Another key issue is flexibility in terms of recruitment; reflecting the fact that recruitment on a seasonal and short-notice basis is required, as well as recruitment for permanent roles (Marine Scotland (1) 2019).

Many communities in Scotland are largely dependent on marine sector economic activity due to their fragile socio-economic nature (Marine Scotland 2020). However, the economic performance of the sector is constrained by restrictive management policies and reduced quotas implemented in response to the declining stocks (Abernethy et al. 2010). The reduction in fishing fleets has directly impacted levels of employment in the affected coastal communities (Marine Scotland 2020). Increase in export of fish could be an economic driver for increased national production and involvement in international trade could bring numerous benefits to the fisheries (Kaimakoudi et al. 2014) and increase in UK and Scottish quota shares over time as a consequence of Brexit may have a positive impact in this regard (Bjørndal and Munro 2020). Henceforth, the Scottish fisheries aim to support fishing and onshore seafood industries of all sizes to grow sustainably, and be internationally competitive, through building and maintaining access to markets. It should be noted that achieving these socio-economic objectives will be possible over a period of time and via long-term investments in the sector. Therefore, improved economic performance is considered as one of the drivers of sustainability in fisheries, and the criteria contributing to the sustainable economic performance of the sector can be described through indicators such as increases in profit, exports and local employment.

#### Biological

Overexploitation of fish resources has been a major problem in the European fisheries and historical data shows a significant loss of biomass of commercially fished demersal fish exploited by the UK fleet (Thurstan et al. 2010). In a UK fisheries audit study by (Guille et al. 2021), six of ‘top-ten’’ stocks identified as economically most important are overfished including the North Sea cod and edible crab and their biomass is below safe biological reference points. Consequently, there is a need for urgent action to eliminate overexploitation of European fisheries and rebuild fish stocks. Furthermore, the incidental catch of non-targeted species, i.e., bycatch, is recognised as one of the main threats to marine fisheries worldwide. In Scotland, the extensive bycatch of juvenile gadoids by the crustacean fishery is thought to jeopardise gadoid stocks (Baudron et al. 2019). Nonetheless, estimating the quantity of bycatch across an entire fishing fleet is challenging due to low observer coverage and a paucity of detailed data on the distribution of fishing effort (Luck et al. 2020). The UK however is the only European country with a long-running, dedicated observer programme for bycatch of protected, endangered, or threatened species (ICES 2018).

Returning living or dead organism to the sea, i.e., discard is also considered a waste of resources and inconsistent with responsible fishing practice, and the accurate documentation of discards is an important issue for fisheries management as it is necessary for the estimation of fishing mortality in stock assessments (Jardim and Fernandes 2013). Discarding is particularly problematic in mixed demersal fisheries where multiple species are caught together which causes issues such losses of fish of commercial value, loss of fisheries mortality data (Witteveen 2019). In Europe, inspired by the environmental concern of discards, the EU imposed the landing obligation (LO) or discard ban since 2019 to end the practice of discarding unwanted catches back to the sea. However, the fishers compliance with the LO depends on market conditions, implementation of the regulation, and enforcement via penalties for non-compliance (Onofri and Maynou 2020). Maintaining a healthy and sustainable stock size is an important objective, which depends on addressing some of the most important problems in this area so as to avoid overexploitation and reduce bycatch and discards.

#### Governance

Brexit has altered the political geography of European fisheries management although the UK fishing industry will still be guided by science-based recommendations of ICES concerning the management of shared stocks in the North Sea (Phillipson and Symes 2018). Following Brexit, as part of the 2020 EU-UK Trade and Cooperation agreement, the UK and the EU, in collaboration with Norway, where relevant, will continue setting TACs for relevant stocks. UK fishermen will have access to EU waters and vice versa. Quota shares will be adjusted over a period up to 2026, with the UK obtaining larger shares for a number of species (Bjørndal and Munro 2020). According to the agreement between the EU and UK, the aspect of zonal attachment has not been directly considered for the initial period of the agreement through to July 2026. During the interim five year “adjustment period”, 25% of EU fishing rights in UK waters will be gradually transferred to the UK fishing fleet, after which yearly negotiations decide whether further adjustments are made.

Furthermore, inclusive governance is an important factor for fisheries. For example, in Europe, the fishers who felt excluded from the decision-making process have been critical of existing management regimes and suggest they can develop more effective systems themselves (Rossiter & Stead, 2003).

In this category, four criteria including the revision in TAC and zonal attachment, transparency, and simplicity of policy measure and inclusive governance have been considered.

#### Logistics

A challenge facing fisheries in some UK areas is an ageing fleet which limits the efficiency and productivity. A significant proportion of the fishing fleet is polluting and over half of the fleet entered service in 1990 or earlier. On the vessel construction supply side, there is limited domestic capacity to meet demand and for vessels over 24 m, i.e., industrial vessels, all production is currently sourced overseas (Marine Scotland 2020). In terms of fleet size in Europe, the industrial fishing vessels fleet, which is dominated by Spain, the Netherlands, and UK, have declined over the years (2000-2017) from 2250 to 1320 units (Nunez-Sanchez et al. 2020).

The energy consumption of vessels mainly depends on the structure and size of the vessels, the engine conditions and use patterns, the fishing gears used, the fishing and trip pattern, the distance to the fishing ground, target species, and their migration routes (Basurko et al. 2013). Given these factors, the increase in efficiency of vessels becomes an important issue since it is directly tied to the economic performance. Therefore, the size, number, and efficiency of the fleet become important factors for the prosperity of the sector since they represent the amount of effort.

#### Environmental

Fisheries leave an environmental footprint on the marine ecosystems in which they operate. The benthic fauna of European continental shelves is a severely impacted community mostly due to intense bottom trawling activities and in Europe, the footprint of bottom impacting fishing on the continental shelf varies between 53-99% per habitat type of the seafloor (down to 200 m) (Jac et al. 2020).

The industrial fishing sector is one of the contributors to global CO_2_ emissions and green house gas emissions from fishing represents a significant part of CO_2_ emissions from world food production (Machado, et al., 2021), yet marine fisheries are typically excluded from global GHG assessment or are generalised based on a limited number of case studies (Parker et al. 2018). The industrial sector is assumed to be exclusively equipped with marine diesel engines due to the deployment of larger sizes of vessels and the heavier, often active gear types (Greer et al. 2019). The carbon footprint of the Scottish small pelagic fleet has been estimated low compared to other aquatic and terrestrial meat, however, the fleet in demersal and shellfish produce much higher CO_2_ levels (Sandison et al. 2021).

Marine pollution is another environmental issue in fisheries. Organisms such as fish, cetaceans, pinnipeds, etc. are negatively affected by the interaction with marine litter via entanglement or ingestions (Consoli et al. 2019).

The environmental impact of fishing activity is captured through the criteria of CO_2_ reduction, reduction in marine pollution (litter), and reduction of impact on seafloor.

### Survey Design

The survey has been designed completely online using Google Forms as the platform. Following the structure suggested by (Raclaw et al. 2020), the survey was organized into:

- The invitation: The invitation to participate in the survey was sent through email to respondents. In the invitation email, an introduction of the project and the purpose of the survey was explained and the survey URL was provided.
- The introduction: The first page of the survey included explanation on the project and additional detail about the survey and how respondents may provide their answers.
- Content modules: This item represented the survey which consisted of a number of criteria and a scale of importance related to each criterion. Five main criteria groups are defined each containing a number of sub criteria (Fig. 1).
- The closing: After the content modules are completed the survey is ended with a feedback question from the respondents. Also agreement statement must have been checked by the respondents that they agree for their response to be used anonymously for research only purposes.

### Question structure

The survey contains both open-ended and closed-ended (fixed choice) questions. Questions that are designed and formatted to obtain yes or no answer, a specific number or piece of information are considered closed questions and any other type which does not ask for a specific answer is considered an open-ended question (Schaeffer and Maynard 2002).

For the qualitative part of this study, open-ended questions were asked from the respondents. The main aim was to understand why the respondent thinks one criterion is (or is not) important in fishery management decision-making. Hence an open-ended question was asked at the end of each section category to which the respondent provided their answers in written format. The open-ended questions are important in collecting more descriptive view points from respondents which are not possible to obtain using only the quantitative scaling system.

For the closed-ended questions, a Likert-type scale ranging from 1–5 (translating to 1 = very low, 2 = low, 3 = moderate, 4 = high, 5 = very high) is defined to demonstrate the level of importance of each criterion in management decision making. The traditional Likert scale format requires participants to indicate their level of agreement or disagreement with survey items on a scale of numbers (e.g., 5 point scale) (Kam 2020). The five point Likert-type system has been used due to practicality, ease of use, and communication with different stakeholders. As some respondents may not be familiar with other methods, a Likert-type scale questionnaire can provide an effective framework for all respondents. The respondents were asked to indicate the level of importance of each of the 16 sub-criteria in decision making (divided in 5 sections) shown in Fig. 1. Provision of response to these multiple-choice questions was mandatory and respondents had to complete all 16 multiple-choice sections to be able to submit the survey.

The closed-ended questions are then followed by an open-ended question which asks the respondent to provide their “opinion/explanation” on why they think each criterion is (or is not) important in decision making. These explanations are carefully read, analysed, and summarised, and the key points are provided in the results section related to each category in Section 3.3.

### Stakeholder groups

Stakeholders are defined as relevant organised group of individuals who can affect and/or be affected by a decision (Banville et al. 1998). Stakeholder engagement in natural resource management is widely accepted as pragmatic and normative good practice and stakeholders are recognised as key players in managing fisheries. Essential steps for developing a suitable sustainability framework have been taken by identifying the end users which are fisheries’ stakeholders including managers, industry representatives, scientists, and fisheries (Crosman et al. 2020) (Krishnaveni and Nandagopal 2018). One of the main aims of this survey is to provide the expert opinions from different groups of stakeholders, therefore the respondents are chosen from diverse backgrounds within the marine fishing sector. The survey was sent to a total number of 74 individuals in the period between August 2020–November 2020. The target group of stakeholders is composed of 29 academics, 10 scientific advisors, 13 industry advisors, and 22 fisheries (producers/vessel owners/skippers).

The respondents in this survey are divided in two main groups:

i. Scientific group: this group consists of academic experts in fisheries management, and scientific advisors, i.e., experts from marine science organisations that deal with the scientific understanding of marine ecosystems.
ii. Industry group: this group consists of industry advisors and representatives, i.e., organisations that promote the interests of Scottish fisheries while ensuring environmental and economic sustainability of the sector, and fisheries i.e., people who are directly involved with fisheries operations, consisting of producer organisations, vessel owners and fishermen.

### Data analysis

One of the goals of this study is to understand different perspectives of the relevant stakeholders with regard to management of fisheries. The aforementioned classification of stakeholders in two groups allows for the elaboration of the importance of management criteria from two viewpoints and deviations between the two groups could be identified which will show the disparity between them.

For computing the score of each criteria, the following approach was taken (Harpe 2015): For each of the 16 criteria, *k*, and overall score of importance, *S*_*k*_, was calculated as the weighted sum of importance scores assigned by respondents. Five levels of importance levels, *i*, were used*:* level 1 = very low, level2 = low, level3 = moderate, level4 = high, level5 = very high. A weighting score *w*_*i*_ = [1–5] was assigned to each level of importance. Finally, the overall score of given criteria, *S*_*k*_, was calculated as the sum of the weighted frequencies across importance levels, divided by the total number of respondents *N*, as shown in Eq. 1:

1 $$S_k = \frac{{\mathop {\sum}\nolimits_{i = 1}^5 {n_{ki} \ast w_i} }}{N}$$

The overall score of a certain criterion could be within the range of 1 ≤ *S*_*k*_ ≤ 5.

## Results

A total number of 20 surveys were received and analysed making the total response rate 27%. The response rate breakdown of each category of respondents is as following: Academic 13% (4/29), Scientific advisory 50% (5/10), Industry advisory 15% (2/13), and Fisheries 40% (9/22). The results of this study have qualitative implications and therefore the sample size in qualitative research tends to be small in order to support the depth of case-oriented analysis. Furthermore, as opposed to probability sampling employed in quantitative research, qualitative samples are purposive, i.e., selected by their capacity to provide richly textured information on the question under investigation (Vasileiou et al. 2018). It is suggested that qualitative studies should generally include between 20-30 interviews and single case studies should generally contain 15 to 30 interviews (Marshall et al. 2015). In the remainder of this section, an analysis of the results is presented.

Table 2 shows the respondents’ details and Table 3 shows the statistical composition of the stakeholders. The Industry group consisted of 47% of the respondents and the scientific group 53% of the respondents. The experts’ levels of experience in the role are segregated into 4 groups where 35% of the respondents have more than 15 years of experience, 15% between 10–15years, 15% between 5–10 years, and 35 % have up to 5 years of experience in their respective roles.

**Table 2:** Participants’ information

Respondent	Gender	Experience	Sector
1	M	Up to 5 years	Academia
2	M	5–10 years	Academia
3	M	Up to 5 years	Academia
4	F	10–15 years	Academia
5	M	More than 15 years	Scientific advisor
6	F	5–10 years	Scientific advisor
7	F	Up to 5 years	Scientific advisor-government executive
8	F	Up to 5 years	Scientific advisor-public/private sector
9	M	5–10 years	Scientific advisor
10	M	Up to 5 years	Industry representative
11	F	Up to 5 years	Marine policy advisory body
12	M	More than 15 years	Fishing association
13	M	10–15 years	Fishing association
14	M	Up to 5 years	Producer
15	M	More than 15 years	Producer
16	M	More than 15 years	Skipper
17	M	More than 15 years	Skipper
18	M	10–15 years	Skipper
19	M	More than 15 years	Vessel Owner
20	M	More than 15 years	Vessel owner

**Table 3:** Composition of the stakeholders

Level of experience in the role	Sector
Up to 5 years = 35%	Scientific group = 45%
5 to 10 years = 15%	
10 To 15 years = 15%	Industry group = 55%
more than 15 years = 35%	

### Scientific group vs Industry group scores

Table 4 presents the results of the comparison of the criteria between two groups. The figures suggest that for the industry group the criteria of local employment, increase in profit, increase in export, increase in number and size of fishing vessels, increase in efficiency of vessels, reducing marine litter, revisions in TAC, and revision in zonal attachments are of higher priority compared to the Scientific group. On the other hand, for the scientific group reducing discards, reducing bycatch, reducing overfishing, inclusive governance, reducing impact on seafloor, and reduction in CO_2_ emissions are of higher priority.

**Table 4:** Comparison of criteria score scientific vs Industry

Criteria		Scientific Group(Sk)	Industry group(Sk)
Socio-economic	Increasing profit	2.8	3.4
	Increasing local employment	2.6	3.5
	Increasing export	2.3	3.2
Biological	Reducing discards	4.2	3.5
	Reducing bycatch	4.2	3.6
	Reducing overfishing	4.3	4.2
Governance	Inclusive governance	4.2	3.8
	Increasing transparency and simplicity of measures	4.1	4.2
	Revision in TAC quota allocations	3.6	3.7
	Revision in zonal attachments	3.4	3.9
Environmental	Reducing fishing impact on seafloor	4.4	3.1
	Reducing marine litter	3.7	4.2
	Reducing CO_2_ emissions from fishing activity	3.6	3.3
Logistics	Increasing the size of fishing vessels	1.7	2.3
	Increasing the number of fishing vessels	1.3	2.6
	Increasing the efficiency of fishing vessels	3.9	4.1

The least amount of disagreement between these two groups is on the increase in transparency and simplicity of policy measures and increase in the efficiency of vessels. Meaning that both groups very closely agree that the policy measures imposed for the management of fisheries shall be more transparent and simple to execute, and that increase in efficiency of vessels in the sector is a high priority.

### Overall criteria scores

Table 5 presents the overall scores-ranked from low importance to high importance- of the sustainability criteria considering a direct average of both groups together (*N* = 20). The figures suggest that among these criteria “Reduction (controlling) in overfishing”, “Inclusive governance”, “Reducing marine litter”, “Increasing transparency and simplicity of measure”, and “Increasing the efficiency of vessels” are considered “highly important” in decision making (S_k_ ≥ 4). “Reducing discards and bycatch”, “Revisions in TAC and zonal attachments”, “Reducing fishing impact on seafloor”, “Reducing CO_2_ emissions”, “Increase in profit and local employment” are considered “moderately important” in decision making (S_k_ < 4). The “Increase in size and number of vessels”, and “Increase in exports” are considered “low importance” in decision making (S_k_ < 3).

**Table 5:** Overall scores of the sustainability indicators

Criteria	Scores	Level of importance
**Increasing the size of fishing vessels**	2	Low
**Increasing the number of fishing vessels**	2.1	Low
**Increasing exports**	2.8	Low
**Increasing profits**	3.1	Moderate
**Increasing local employment**	3.1	Moderate
**Reducing CO2 emissions from fishing activity**	3.4	Moderate
**Revision in TAC quota allocations**	3.7	Moderate
**Revision in zonal attachments**	3.7	Moderate
**Reducing fishing impact on seafloor**	3.7	Moderate
**Reducing discards**	3.8	Moderate
**Reducing bycatch**	3.9	Moderate
**Increasing the efficiency of fishing vessels**	4	High
**Inclusive governance**	4	High
**Reducing marine litter**	4	High
**Increasing transparency and simplicity of measures**	4.2	High
**Reducing overfishing**	4.3	High

### Analysis of open-ended questions

In this section, an analysis of the answers to open-ended questions that were asked for each category is presented. Following the format of the previous section the written responses are categorised into Scientific group and Industry group and present the results related to each category group in different sections. The tables present the summary of the opinions made by expert and are classified based on the particular sub-criteria.

#### Socio-economic

The industry group almost uniformly agreed that the socio-economic aspects, in particular increase in profit are important for the sustainability of fisheries and an important driver for the long-term success of fisheries. Between the scientific advisory groups and academia different views exist about the role of socio-economic factors on the sustainability of fisheries. While most agreed that the socio-economic criteria are important, some disagreed with the role of these criteria in driving the sustainability of the sector while also emphasising the balance of socio-economic and ecological factors. Table 6 presents a summary of the respondents’ views on each criteria.

**Table 6:** Experts opinions on socio-economic criteria

Criteria	Industry Group	Scientific group
Profit	Fishing association (1): “*The profitability of the business is the key to long-term success*”. Producer (2): “*Long term sustainability of stocks will flow from long term industry investment which provides customers with a stable supply of product* Fishing association (2): “*Everything is growing more expensive so profits going up, helps that*”. Industry advisory (1): “*Increasing profit and local employment serve the need of keeping the activities thriving and attract people into the sector. Fisheries are historically very important for coastal communities and helped to build them as they are now*” *so profits up would help that*”. Skipper (1): “*Not worth being here if operating at a loss*”. Skipper (2): “*All of the criteria just need improvement*”.	Scientific advisory (1): “*Each (profit, employment, export) are important criterion but should not individually be the main driver*”. Scientific advisory (2): “*Without real profitability and jobs the fishery cannot prosper. But that cannot come at the expense of the ecosystem, because that will reduce long term profitability of the fishery*”. Academic (1): “*The profit and employment criteria have to be balanced with each other to maintain overall economic and social sustainability*”. Academic (2): “*Profit ultimately sustains the operation of the fishery and an increase allows for longer term planning*”. Scientific advisory (3): “*None of these drive fisheries management decision making, the key issue is minimum sustainable whinge*”. Scientific advisory (4): “*Increasing profit does not often equate with sustainability*”.
Employment	Fishing association (1): “*Employment strategy is the focus of government*”. Producer (1): “*The catching sector should be supporting the local fishing communities as they are temporary custodians of the fishery resources and they rely on the onshore sector to support their activities*”. Fishing association (2): “*Employment, attracts more people so less poverty so more jobs*”.	Academic (2): “*Increasing local employment maintains and strengthens the link between the fishery and the community which drives sustainability*”.
Export	Fishing association (1): “*The export strategy of the company is created with an eye on profitability*”. Producer (1): “*We have no direct interest in exporting*”. Fishing association (2): “*The world’s demand for fish is growing increase exports are needed to meet this demand*”.	Academic (1): “*Increasing export is more problematical-e.g., many regional fisheries supply UK markets to varying degrees. There are substantial sub-regional variations in terms of species caught, technology (boat and gear), local and export markets, levels of direct employment, relationship with the shore industry, notably procession and transport, and overall location which reflects the inter-relationships of these factors*”. Academic (2): “*Increasing exports grows the market for produce but an overreliance brings its own problems*”.

#### Biological

Different views were expressed within the industry group’s respondents regarding overfishing, bycatch, and discard. While there is consensus that overfishing leads to the breakdown of stocks, and is a high priority for the industry to be controlled and avoided, there were various opinions on reductions in bycatch and discards. Some fishermen did not view bycatch as negative, especially when the bycatch was simply non-targeted and as long as quota is available for it and one of the fishermen perceived discard as important for feeding marine ecosystem rather than waste. The scientific group maintained that limiting overfishing is key for the sustainability of the sector, however reduction in bycatch was considered as not entirely possible and one of the respondents maintained that if quota allocations are provided for bycatch, there is no need to reduce it especially in mixed demersal fisheries were bycatch may consist a large share of the catch. Table 7 presents a summary of the respondents’ views on each criteria.

**Table 7:** Experts opinions on Biological criteria

Criteria	Industry	Academia
**Reduction in overfishing**	Fishing association (1)*: “Maintaining fishing mortality at or below maximum sustainable yield is important for the long-term prosperity of the stocks and the fishing business”.* Industry advisory (1): *“Reducing overfishing is high in everyone’s agenda since the fisheries rely on the presence of fish in the future and sustainability is paramount when looking into the future”*. Owner/Skipper (1): *“Lets think 25 years down the line for us and another for our next generation to make this a viable job with a viable future”.* Producer (2): *“overfishing leads to the breakdown of stocks, instability of supply, loss of market and thus loss of investment. That loss of investment exposes fisheries to short term profit drivers which act to hinder attempts at stock recovery”.*	Academic (1)*: “Reduction of all three should be a high priority given the observable pressure on the fish stocks concerned*”. Scientific advisory (2): “Without reducing overfishing, bycatch and discard the fishery cannot be sustainable”. Scientific advisory (4): *“Measures to limit and reduce discard, bycatch, and overfishing lead to more sustainable fisheries and all three are playing a role in reducing MSY”.*
**Reduction in discards and bycatch**	Industry advisory (1): *“The reduction of the discard is a priority because discarding fish does not benefit anyone, neither the fishermen wasting good fish and time spent at sea nor the stock that is affected. The reduction of bycatch depends on its definition. If we are talking about protected species then of course priority needs to be high, but in the definition of bycatch often other commercial, non-target species are included and in this case, assuming the quota is available for them, they could constitute an important component of the catch”.* Fishing association (1): *“Discarding is a symptom of unwanted catch or bycatch. The important issues are to avoid species or fish size you don’t want”.* Producer (1)*:“Reductions in discards and unwanted by-catches in the demersal mixed fishery is important to us by has become something of a “holy grail”. We would judge that the elimination of discards and unwanted bycatch is impossible. There is no need to reduce by-catch if there is a quota allocation for it and a mixed demersal fishery comprises many bycatches”.* Producer (2)*: “Reducing bycatch, overfishing and discards are all important for preserving biodiversity”.* Skipper (2): *“Discard is not a waste and is an important part of feeding the marine eco-systems*”.	Scientific advisory (1): *“Bycatch and discards are increasing and effective compliance and monitoring will reduce discarding more than biological management inputs”.* Academic (2): *“Discards are both unprofitable and distort the data on mortality and overfishing harms the long term sustainability of fisheries”*.

#### Governance

The industry held strong views on the transparency of policy measures, inclusive governance, and co-management approaches were suggested to provide realistic and workable policies and to increase fishermen’s compliance and accountability and some skippers were pessimistic about the policy measures implemented. Furthermore, regular revision of allocation of quota was important to the industry to take account of the changes in spatial distribution of stock and provide a sustainable balance.

The Scientific group suggested that [some stock assessment] use outdated data and a review of quota allocation is necessary. Additionally, adopting an inclusive governance approach, and inclusive governance was suggested to be encouraging fishers to follow and comply with the regulations. A summary of the respondents’ views is presented in Table 8.

**Table 8:** Experts opinions on Governance criteria

Criteria	Industry	Academia
**Inclusive governance**	Industry advisory (1)*: “Inclusive governance instead of top-down approaches, leads to writing policies which are more realistic and workable. Co-management provides a shared ownership of the regulations which makes it easier for the fishermen to comply and being accountable for their activities”.* Fishing association (1): *“Governance and transparency for me go hand in hand. The closer that those being managed are to the managers, preferable co-management, then the more positive response there will be with regard to legitimacy of regulation compliance”.* Skipper (1): *“We need governance otherwise it will fall down. Rules need to be universalised within reason. Stop the fat cat taking control or there’s no hope for future generations”.* Skipper (2): *“The system as it is needs overhauling as people making decisions have not a clue about how the job is or how it works”.* Producer (1): *“All sectors of the fishing industry must be part of the governance process in order for it to be successful”.* Fishing association (2): “*governance so that everyone is treated equally regardless of where they work”.*	Academia (2)*: “Inclusive governance, increased transparency, and simplicity of measure are likely to lead to better adoption and compliance”.*
**Revisions in TAC and zonal attachment**	Fishing association (1): *“Issues such as quota shares, zonal attachments etc should be kept under routine review”.* Producer (2): *“Revision of zonal attachment should reduce over quota discards as biologically sensible levels of quota will be available for all stocks caught in a mixed fishery. Other criteria are political and their desirability is determined by socio-economic objectives in fisheries policies”.* Producer (1): *“The way quotas are allocated must be constantly reviewed in order to take full account of change to fish stocks’ spatial distribution*”*;* Fishing association (2): *“Quota allocations should be reviewed since it is disproportionate in catch areas. Zonal attachment recognised as there needs to be a balance between maintaining biodiversity and livelihoods in the economy”.*	.Academia (2) “*TAC quota allocations are often based on outdated data and need be kept under review. Review of zonal attachments can help the function of the fishery but too much change can lead to conflict between nations and regions”.* Academia (1): *“All four governance criteria are high priority. Quota allocations and zonal attachments belong to the category of technical management measures upon which transparency and governance ultimately depends*”*.*
**Transparency and simplicity of measures**	Producer (1): *“over the years measure have become complicated because they have been subjected to the “sticking plaster” approach whereas the core issues have not been tackled”.* Fishing association (2): *“Transparency is needed so it makes everything easier to understand”*.	Scientific advisory (2): *“Transparent and simple measures are important as are inclusive governance. If you can include fishers in the process of making decisions, they will follow the decisions”.* Scientific advisory (4): *“Transparency increases trust in measures and acceptance of solutions”.*

#### Environmental

The majority of respondents in industry group showed a positive view on the need to control the environmental impacts of fishing and reducing impact on seafloor, reduction in marine pollution, and reduction in CO_2_. However, some skippers did not think that the impact on seabed is negative and argued that the seabed could recover quickly. Also, the CO_2_ emissions were considered to be low compared to other sectors (Table 9). The Scientific group maintained that all three criteria are important for maintaining a healthy ecosystem and stock although they expressed some uncertainties on the impact of marine pollution and significance of fishery sector CO_2_ emissions. The marine pollution mainly refers to the anthropogenic litter, more specifically any persistent, manufactured or processed solid material discarded, disposed or abandoned in the marine and coastal environment (Int-Veen, et al., 2021). One of the main contributors to marine plastic pollution is the fishing industry. In the North Sea, plastic is the main pollutant and fishing originated litter represent the highest portion of that (Buhl-Mortensen & Buhl-Mortensen, 2017).

**Table 9:** Experts opinions on Environmental criteria

Criteria	Industry	Academia
**Reducing CO**_**2**_	Fishing association (1)*: “Reducing the emissions in fishing industry is less of an issue given the already low carbon footprint of the sector compared to other protein delivering sectors”.* Fishing association (2)*: “CO*_*2*_ *emissions need to be reduced to combat global warming”.* Producer (1): *“All these issues are important to us but we must maintain a profitable catching sector if the industry and our fishing communities is to have a future”.* Skipper (1): *“The fishing industry emission is a very low impact, especially compared to other industries (the wind and oil have higher emissions). We will get there but need time for fleet to modernise because it is very expensive”.*	Scientific advisory (2)*: “All of these criteria (reduction of CO*_*2*_*, impact on sea bed and marine litter) are important in their own right”.* Academic (2)*: “Reducing CO*_*2*_ *is important but while global CO*_*2*_ *levels and their effects are important to the fishery, fishing itself is not a leading contributor*”.
**Reducing marine litter**	Fishing association (2): *“Pollution is increasing so maritime litter would need to be reduced”.* Producer (2):*“All elements should feature in a fisheries policy. The importance of reducing fishing impact on sea floor varies with geography/substrate, the other two points (emissions and litter) are global in scope”.* Owner/Skipper (1): *“Litter is a no no, I’m now taking rubbish back to shore for 22 years and we are still catching rubbish but it is not that all fishermen dump rubbish!*”.	Academic (1)*:* “*All three [criteria] are high priority, although solutions will require long-term application to become effective”.* Academic (2): “*Reducing marine litter are important but the direct impact of marine litter on the fishery is currently uncertain”.*
**Reducing impact on seafloor**	Fishing association (1): *“It is important that we continue to improve our fishing techniques so that our disturbance of the seabed and seabed features is reduced to the minimum. It is also extremely important that we treat the sea as our own garden which we like to see in great condition (most of us anyhow)”.* Fishing association (2)*: “Ocean needs to stay healthy since food chain in ocean relies on seafloor*”. Owner/skipper (1): *“The seabed is low impacted and where it is it does it good to turn it over like a farmer ploughing a field, it goes stagnant if not and the evidence is there”.* Skipper (2): *“The seafloor recovers extremely quickly due to the effects of wind and tide”.*	Academic (2): *“The seafloor habitat being considered as part of an ecosystem-based approach to fisheries management is crucial, especially in a demersal fishery”.* Scientific Advisory (3): *“Impact [of fisheries] on seabed is increasing”.* Scientific advisory (4)*:* “*Environmental protection measures are crucial to maintaining a healthy ecosystem and healthy abundant fish stock”.*

#### Logistics

The industry group maintained that while the increase in number and size of vessel is not a determining factor, increasing the efficiency of vessels is an important criterion for sustainability of fisheries. Improvements in efficiency could lead to better selectivity, improved competitiveness of UK’s fleet and would lead to savings in time and energy and vessels safety was also pointed out as a factor for improved fleet efficiency. Similar to the Industry group, the Scientific group maintain that the efficiency of vessels in terms of targeting the right species is important criteria (Table 10).

**Table 10:** Experts opinions on Logistics criteria

Criteria	Industry	Academia
**Increase in efficiency of fleet**	Fishing association (1): *“If a fishery is properly managed then the size or numbers of vessels is irrelevant and become a political decision based on social criteria. Efficiency of vessels is an important aspect in that technical creep can have a negative impact on mortality if the number and size of the vessels remains a constant”. “Efficiency increases profit and reduces effort”.* Producer (2): “*The most important criteria is to match fishing capacity with fishing opportunities. From a long term perspective, efficient harvesting would allow UK seafood to remain competitive on the global market. However, increasing efficiency in an effort based management system can lead to overfishing. Increase efficiency is desirable in a TAC and quota regime”.* Producer (1): “*Vessels need to be safe to withstand the increasing amount of storms being experiences and their strength. fishing vessels and their efficiency (the fishing capacity) should match the amount of fish that can be removed by fishing: because this will vary we must guard against creating over-capacity in the fleet”.* Fishing association (2):“*If you can fish efficiently, there is no need to increase the size of your vessel. There is plenty of vessels just now for what they are allowed to catch. Increasing efficiency means less time and energy is wasted”.* Skipper(2):“*Efficiency increases profit so reduces effort”.*	Scientific advisory (1): “*Efficiency is important in terms of targeted, selective fishing rather than volume”.* Scientific advisory (4): *“There need to analysis of the trade-offs between vessel size, number and efficiency in terms of contribution to environmental protection and fish stock sustainability. More vessels will lead to more catch, larger vessels lead to fewer jobs”.* Academic (1): “*While efficiency is related to both size and number of fishing vessels, it should still be high priority”.*
**Increase in size and number of fleet**	Industry advisory (1)*: “There are no absolute answers, the fleet segment and the various fishing activities around the coast of Scotland for example, require different classes of vessels and a variety of dimension and performances which cannot be easily simplified”.* Owner/skipper (1): *“The fleets are good size and supply can meet demand. The fleet does need modernising, but the whole Europe should ban multi-rig before it ruins the market”.* Producer (2): *“Increasing size or number of vessels very much depends upon whether one prioritises economic return or maintenance of employment in the catching sector (though not necessarily in the processing sector)”.*	Academic (1)*: “Fisheries incorporate a number of different size categories which vary regionally and with regard to individual ports at which there are based. In general increasing size and number of vessels is low priority under present circumstances”.* Scientific advisory (2): *“More vessels will lead to more catch. Larger vessels lead to less jobs”.* Academic (2)*: “Increasing the size and number of vessels should not be a goal. It should be monitored and any changes factored into decision making. Increasing the efficiency of the fleet should be a focus as it will improve the profitability but include measures to improve the efficiency with which catch species are targeted”.*

## Discussion

The results of this paper provide an original contribution to the literature on sustainable management of fisheries by developing a sustainability framework for Scottish fisheries using a multitude of criteria. Sottish fisheries are the centre of UK’s fishery sector and have been facing serious challenges such as overexploitation of key stocks, change of governance structure following Brexit, decline in employment as well as marine pollution.

By comparing and contrasting viewpoints of the industry vs scientific advisory groups, this study shows interesting results about the key priorities for the sustainable management of fisheries in Scottish fisheries. The results of the study show the industry group ranks the socio-economic category (inc. exports, employment, profit) higher in terms of importance in decision making, compared to the scientific group. Fishermen and producers expressed that increase in profit is key to long-term success of the fishery and that fishery sector should support the local community. This shows that the industry group perceives the economic performance as one of the drivers for the sustainability of their sector while the scientific group does not consider it as important as the former group in policy making. This misalignment in perception may sometimes lead to implementation of policies that are perceived to harm the sector in terms of profitability. These results are in line with the arguments in the literature that social objectives could be lost in multi-level governance systems of fisheries (Symes and Philipson 2009). Emphasis on social objectives is particularly important for fishery-dependent communities such as Fraserburgh in Scotland where 15% of its workforce is involved in the fishing industry (Ross 2013).

In the Biological category, the scientific group ranks the reduction of discards and bycatch highly important while the industry gives it moderate importance and some fishermen do not think discarding is wasteful and that bycatch is not a negative issue that should be reduced although some studies in the literature shows that bycatch of juvenile gadoids have impacted the gadoid stock in the Scottish fisheries (Baudron et al. 2019). It should be noted that The statements recorded are much more about bycatch in terms of non-targeted stocks or endangered species rather than of immature individuals of the target stock. Even the scientist group did not make specific reference to the discarding of undersized, immature fish of the target stock. This could be due to the fact that in a multi-species fishery bycatch is unavoidable and if proper quota is allocated they could part a significant share of the catch.

In the environmental category, the scientific group places high importance on reducing the impact of fishing on seabed, while the industry group does not, and some fishermen (as explained in the qualitative analysis section) do not perceive that the impact on seabed is significant. This difference in viewpoints may lead to fishing practices impacting the seafloor and therefore promoting awareness among the industry is crucial to minimise the negative environmental impact of fishing. For the CO_2_ reduction both groups have considered it moderately important and most participants from both group acknowledged that it should be reduced, although some fishermen did not agree that CO_2_ generated by the fishing industry is a particular problem. Nonetheless, studies show that CO_2_ emissions from global marine fisheries maybe considerable higher than previously suggested and fishing sector is a contributor to global emissions (Greer et al. 2019).

In the governance category, the industry group places higher importance on the revisions of TAC and zonal attachment compared to the scientific group. As reflected in the industry responses, regular and routine revisions of the quota allocations is fundamental in order to take into account the changes in the spatial distribution of fish and keep the balance in terms of biodiversity and catch. This issue is particularly important for Scottish fisheries since following Brexit, the fisheries management regimes may be shifted to comply with the new regulations

The results also show convergences between the two groups. Both groups in this study indicate that increasing the efficiency of fishing vessels to improve the selectivity of catch as well as improving the operations is of high importance; both groups also agree that increasing the transparency and simplicity of policy measures is highly important for decision making. Both groups pointed out the need for co-management approaches, inclusive governance rather than top-down approaches, current complex measures, and the ineffectiveness of the “sticking patches” approach to alleviate core problems.

From a policy perspective, this survey provides valuable insights from both groups (industry and scientific) which could help direct the decision-makers in determining enhanced policies for sustainable management of Scottish fisheries. This indicates that industry, as well as the scientific community, are aware of the dangers of overexploitation that threaten the long term sustainability of the industry; however, increased transparency and simplicity of measures and co-management is required in order for the management policies for maintaining a healthy stock size level to be effectively executed. In terms of environmental impact, reduction of marine litter is considered highly important amongst both groups, which raises awareness about the global marine pollution endangering the marine ecosystem and the problems faced by the fishery industry in terms of their catch levels. Lastly, the high importance level associated with the need for increased efficiency of vessels signals the necessity for investment and improvements in the fleet. Modernisation of the fleet (alternative renewable fuel sources, improved engine design) and gear technologies (e.g., reduced gear drag and changing from active to passive gear techniques) could result in lower fuel consumption leading to improved economic performance, especially since more than half of all vessels in UK fleet were built before 1990 (MMO 2019).

## Conclusions

In this paper further evidence and confirmation are provided on the sustainability factors that are important for decision making through gathering primary data from UK’s North Sea Scottish Fisheries. The suggested sustainability framework considers aspects related to economic growth, social development, governance and policy, biology, environment, and logistics of the marine fisheries. The key management priorities revealed in this survey show that for both the scientific and industry groups, controlling (reducing) overfishing has the highest priority score in comparison to all other criteria, followed by increasing the transparency and simplicity of policy measures, inclusive governance, increasing the efficiency of vessels and reducing marine pollution.

Scottish fisheries are home to the largest fish operators in the UK but also share stocks within the wider North Sea region. UK and EU share over 100 stocks and 80% of the ‘top-ten’’ economically valuable stocks are shared with mainly the EU and are subject to TAC (Marine Scotland 2020). Therefore, the management objectives that are highlighted via this framework could be important for other European fisheries in the North Sea due to the existing interrelations between the UK and European fisheries sectors and their sustainable management is not only important for the UK but it impacts other EU countries in the North Sea region. The results of this study show that sustainable management paradigms for these fisheries following Brexit require a greater range of objectives to be considered into the decisional framework and should not focus only on biological goals. These objectives may be conflicting sometimes, for example for achieving sustainable levels of catch, the socio-economic objectives may be compromised affecting the long-term investments in this sector. Hence such participatory frameworks that are co-developed with the industry stakeholders and fisheries, would help decision-makers in setting the sustainability goals which are better aligned with all stakeholders’ objectives.

However, the implementation of such holistic approaches has proven to be difficult. The scientific advice has to move from mainly biological towards integrating socio-economic aspects with varying degrees of stakeholder involvement from consultation to stronger collaborations (Mackinson et al. 2011). Building trust between policy makers and fisheries through transparency and collaborative decision-making is increasingly important for commercial fisheries to operate effectively. The contribution of this study is providing a multi-criteria sustainability framework through (i) collecting and analysing primary quantitative and qualitative data gathered from a diverse group of stakeholders in the Scottish fishery sector and scientific community, (ii) prioritising a diverse range of criteria in terms of importance in decision making from industry and scientific community perspective, and (iii) elaboration of the key management objectives within the context of sustainable management of fisheries. This study provides insight for the Scottish fisheries sector, environmental protection groups, and scientific advisory groups for the enhanced implementation of fisheries policies. At the time of writing this paper, there is significant political uncertainty in the UK’s fishery sector with ongoing negotiations on fisheries rights and relations with the EU. This reform will undoubtedly affect the fisheries sector in UK sharing the North Sea with other EU members. Therefore, it is suggested that this aspect is monitored as political development regarding Brexit unfolds. The research on the reformed fisheries management following UK’s exit, may become an important research avenue that could be investigated by researchers in the marine sector.
